# On modeling of coronavirus-19 disease under Mittag-Leffler power law

**DOI:** 10.1186/s13662-020-02943-z

**Published:** 2020-09-11

**Authors:** Samia Bushnaq, Kamal Shah, Hussam Alrabaiah

**Affiliations:** 1 Department of Basic Sciences, King Abdullah II for Engineering, Princess Sumaya University for Technology, Amman, 11941 Jordan; 2 Department of Mathematics, University of Malakand, Dir(L), 18000 Khyber Pakhtunkhwa, Pakistan; 3 Al Ain University, Al Ain, UAE; 4 Mathematics Department, Tafila Technical University, Tafila, Jordan

**Keywords:** 26A33, 34A08, 93A30, Coronavirus disease model, $(\mathcal{ABC})$ derivative, Stability results, Fractional Adams–Bashforth (AB) method, Numerical simulations

## Abstract

This paper investigates a new model on coronavirus-19 disease (COVID-19) with three compartments including susceptible, infected, and recovered class under Mittag-Leffler type derivative. The mentioned derivative has been introduced by Atangana, Baleanu, and Caputo abbreviated as $(\mathcal{ABC})$. Upon utilizing fixed point theory, we first prove the existence of at least one solution for the considered model and its uniqueness. Also, some results about stability of Ulam–Hyers type are also established. By applying a numerical technique called fractional Adams–Bashforth (AB) method, we develop a scheme for the approximate solutions to the considered model. Using some real available data, we perform the concerned numerical simulation corresponding to different values of fractional order.

## Introduction

In present time a dangerous pandemic known as COVID-19 has been widely transmitted in the whole world. This is the seventh generation of coronavirus and therefore researchers have named it COVID-19. Nearly 7 millions people have been infected with the virus all over the world, and 0.4 million have been pushed to death in almost 180 countries of the world. Many countries of the world have ordered to lockdown the cites and to stop the air as well as plane traffic so that the infection may be controlled from further spreading. WHO announced it a global pandemic [1]. The economic situation of many countries, as well as health system of several countries, is near to collapse. Historically, at the end of 2019, the mentioned outbreak started from a seafood market of Wuhan city, and within a month the whole city was attacked by the virus. The Chinese government lockdowned the whole city in time, and the infected people, which they called the quarantined people, were separated; in this way the mentioned state after two months was able to control the infection in their country. On the other hand, due to immigration and traveling the infection was transmitted in two months in almost all countries of the world. Therefore researchers, physicians, and policy makers have started working day and night to control this killer infection from further spreading. Each country has taken their own precautionary measures, for details, see [2–5].

Different techniques, procedures, and tools in the past were adopted to understand and to make some some precautionary measures during such outbreaks. Therefore we know that mathematical models are powerful tools to understand the transmission dynamics of infectious diseases and to make future planing. In this regards large numbers of infectious models corresponding to various infectious diseases in history have been developed, we refer to a few [6–9]. Infectious diseases are a massive threat to humanity and can greatly affect the economy of a state. Proper understanding of disease dynamics could play an important role in the elimination of infection from the community. Further, the implementation of suitable control strategies against the disease transmission was assumed to be a big challenge. The approach of mathematical modeling is one of the key tools for handling such and other challenges. A number of general and disease models have been investigated in the existing literature, which enables us to explore and control the spread of infectious diseases in a better way [10–12]. Also the aforementioned model has been studied under various incidence rates including concave, linear, and nonlinear incidence rate. Each rate has its own importance, see for detail [13, 14]. But investigation of biological models under convex incidence rates is more informative as in such investigation a convex function of the infected class under double exposure is taken. Such involvement of double exposure helps more in the spreading of infection, and its dynamics is more powerful in forming the control procedure [15, 16].

The above-mentioned epidemic models as well as many others in the literature are actually based on integer-order differential equations (IDEs). However, in the last few years, it has been noticed that with the help of fractional-order differential equations (FDEs) one can model a universal phenomenon with greater degree of freedom [17]. This idea was implemented in many fields including engineering, economics, control theory, finance, and some up to the mark results were founded. Fractional calculus is the generalization of classical integer-order calculus. The increasing interest of using FDEs in the modeling of complex real world problems is due to their various properties which could not be found in IDEs. In contrast to IDEs, which are local in nature, FDEs are nonlocal and possess the memory effects which make them superior as in many situations the future state of the model depends not only upon the current state but also on the previous history. These features enable FDEs to effectively model the phenomenon having not only the non-Gaussian but also for non-Markovian behavior. Further, the classical IDEs are unable to provide the information in between two different integer values, and it can be made clear with the help of FDEs. Various types of fractional-order operators were introduced in the existing literature to overcome such limitations of integer-order derivative. The applications of these fractional operators can be found in various fields [18–22].

In the eighteenth century Riemann, Liouville, Euler, and Fourier were struggling in producing significant results in ordinary calculus. At the same time, great contributions were made in the area of fractional calculus. This is due to the various applications of fractional calculus in the field of mathematical modeling where several hereditary materials and memory processes cannot be explained clearly by ordinary calculus. Because fractional calculus, which includes classical calculus, is a special case, it has greater degree of freedom in differential operator as compared to ordinary differential operator which is local in nature. The important applications of the said calculus may be found in [23–28]. Therefore, researchers have given very much attention to studying of fractional derivatives and integrals. In fact, fractional derivative is a definite integral which geometrically interprets the accumulation of the whole function or the whole spectrum which globalizes it. In investigation of differential equations for qualitative, numerical studies and optimization, significant contributions have been made by researchers, we refer to a few [29–32]. It is also remarkable that fractional differential operators have been defined in a number of ways. It is a well-known fact that the definite integral has no regular kernel, therefore both types of kernels have been involved in various definitions. One of the important definitions which have very recently attracted the attention is the $\mathcal{ABC}$ derivative introduced by Atangana, Baleanu, and Caputo [33] in 2016. The mentioned derivative exhibits the singular kernel by nonsingular kernel and therefore has been greatly studied [34–36]. Since most of the nonlinear problems are difficult to solve for their exact or analytical solution, various numerical procedures have been established to solve the mentioned problems, we refer to [37–39]. Recently the said numerical methods have been extended to investigate FDEs under the $\mathcal{ABC}$ derivative, see [40, 41].

Due to the current situation, many studies have been recorded on mathematical modeling of the outbreak of COVID-19, we refer to a few [42–48]. Currently this area of mathematical models for the coronavirus infectious diseases is a warm area of research. Therefore we consider the following model of three compartments, including the susceptible population $P(t)$, the infected papulation $I(t)$, and the removed class $R(t)$ (death due to corona or natural) at time *t* for $\kappa \in (0, 1]$ as follows:

1 $$ \textstyle\begin{cases} {}^{\mathcal{ABC}}\mathrm{D}_{+0}^{\kappa }P(t)=a -\sigma I(t)P(t)(1+ \gamma I(t)) -dP(t) +\alpha R(t), \\ {}^{\mathcal{ABC}}\mathrm{D}_{+0}^{\kappa }I(t)=\sigma I(t)P(t)(1+ \gamma I(t))-(\mu +d+\delta -b)I(t), \\ {}^{\mathcal{ABC}}\mathrm{D}_{+0}^{\kappa } R(t)=\mu I(t) -(\alpha +d)R(t), \end{cases} $$

with given conditions

$$ P(0)=P_{0}, \quad\quad I(0)=I_{0}, \quad\quad R(0)=R_{0}. $$

Keep in mind that the right-hand sides of model (1) vanish at $t=0$ as already proved in [49]. The parameters involved in model (1) are described as in Table 1. Some necessary assumptions that we impose on the model is that all the parameters involved in model (1) are nonnegative. The above model is investigated from three different aspects. First as the given model (1) is newly formulated, we establish its existence by using fixed point theory. On the other hand, stability is important, so we are going to investigate Hyers–Ulam type stability for the concerned model. For the general models using $\mathcal{ABC}$ derivative of fractional order, the mentioned two aspects have been investigated, we refer to [50–53]. Keep in mind that the right-hand sides of the above model vanish at zero.

**Table 1:** Illustration of the parameters involved in model (1)

Parameters	The physical interpretation
*P*(*t*)	Susceptible compartment
*I*(*t*)	Infected compartment
*R*(*t*)	Removed compartment due to death by infection or natural
*a*	The recruitment rate
*d*	Natural death
*δ*	Death due to corona
*b*	The immigration rate of infected individuals
*μ*	Infected population goes to recovered
*σ*	The infection rate
*γ*	The rate at which the recovered individuals lose immunity
*α*	The recovery rate

Further the involved state functions of the model obey $\mathbf{N}(t)=P(t)+I(t)+R(t)$, where the total population is **N**. For nonlinear problems it is always difficult to find their exact solution. Therefore, various numerical procedures (methods) have been constructed in literature to deal with such problems, see [54–56]. For classical and usual fractional derivatives, the numerical schemes have been framed, and on further slight modification they may be extended to the new nonlocal FODEs, see [57–63]. Therefore a fractional-type two-step AB method is applied to simulate the results via Matlab-16. Also it is natural that the model we investigate exists in the real world, for this purpose, numerous fixed point theories were developed in past. Here to derive the required needs for the considered model, we will use Banach and Krasnoselskii-type theorem to establish some adequate results for the existence of at least one solution. Also stability is needed in respect to numerical solution, so we attempt on Ulam-type stability for the considered model. The mentioned stability has been investigated for the usual fractional derivatives in extensive research work; however, the same has not been investigated for $\mathcal{ABC}$ derivatives. Finally, the results are displayed against the real data which have been taken from the source [64].

## Fundamental results

Here some basic materials are taken from [30–32].

### Definition 2.1

If $x(t) \in \mathcal{H}^{1}(0, \tau )$ and $r \in (0, 1]$, then the $\mathcal{ABC}$ derivative is defined using Mittag-Leffler function $\mathcal{M}_{\kappa }$ as follows:

2 $$\begin{aligned} {}^{\mathcal{ABC}}\mathrm{D}_{+0}^{\kappa }x(t)= \frac{\mathcal{ABC}(\kappa )}{1-\kappa } \int _{0}^{t}\frac{d}{d\theta }x( \theta ) \mathcal{M}_{\kappa } \biggl[\frac{-\kappa }{1-\kappa } (t- \theta )^{\kappa } \biggr]\,d\theta . \end{aligned}$$

Here $\mathcal{ABC}(\kappa )$ is known as a normalization function which is defined as $\mathcal{ABC}(0)=\mathcal{ABC}(1)=1$.

### Definition 2.2

Let $x\in L[0, T]$, the fractional integral in the Atangana–Baleanu sense is defined by

3 $$\begin{aligned} {}^{\mathcal{ABC}}\mathbf{I}_{+0}^{\kappa }x(t)= \frac{1-\kappa }{\mathcal{ABC}(\kappa )}x(t)+ \frac{\kappa }{\mathcal{ABC}(\kappa ) \Gamma (\kappa )} \int _{0}^{t} (t- \theta )^{\kappa -1}x(\theta ) \,d\theta . \end{aligned}$$

### Lemma 2.3

(Theorem 3 of [50])

*The solution of the given problem for* $\kappa \in (0, 1]$

$$\begin{aligned}& {}^{\mathcal{ABC}}\mathrm{D}_{0}^{\kappa }x(t)=z(t), \quad t\in [0, \tau ], \\& x(0)=x_{0}, \end{aligned}$$

*is provided by*

$$ x(t)=x_{0}+\frac{(1-\kappa )}{\mathcal{ABC}(\kappa )}z(t)+ \frac{\kappa }{\Gamma (\kappa )\mathcal{ABC}(\kappa )} \int _{0}^{t} (t- \theta )^{\kappa -1}z(\theta ) \,d\theta . $$

### Note

For the qualitative analysis, let $0\leq t\leq \tau <\infty $ and denote $\mathcal{J}=[0, \tau ]$, we define Banach space $\mathbf{Z}=\mathcal{J}\times \mathbb{R}^{3}\rightarrow \mathbb{R}$ under the norm $\Vert W \Vert = \Vert (P, I,R) \Vert =\max_{t\in \mathcal{J}}[ \vert P(t) \vert + \vert I(t) \vert + \vert R(t) \vert ]$.

### Theorem 2.4

([52])

*“Let* **B** *be a convex subset of* **Z** *and assume that* **F**, **G** *are two operators with*

1. $\mathbf{F}w + \mathbf{G}w\in \mathbf{B}$*for every* $w\in \mathbf{B}$;
2. **F** *is a contraction*;
3. **G** *is continuous and compact*.

*Then the operator equation* $\mathbf{F}w+\mathbf{G}w=w$*has at least one solution*.*”*

## Qualitative analysis of the considered model

Here first of all we find out a feasible region for the solution and its boundedness under integer order derivative in the given theorem.

### Theorem 3.1

*Model* (1) *under consideration is bounded in the feasible region given by*

$$ \mathbf{S}= \biggl\{ (P, I, R) \in \mathbb{R}^{3}_{+}: 0\leq \mathbf{N}(t)\leq \frac{a}{d-b} \biggr\} , \quad \textit{where } d>b. $$

### Proof

As we have $\mathbf{N}(t)=p(t)+I(t)+R(t)$, therefore adding all three equations of model (1) and taking integer order derivative, we have

4 $$\begin{aligned} \dot{\mathbf{N}}(t) =&a-d(P+I+R)-\delta I+b I \\ \leq & a-d(P+I+R)+bI \\ \leq &a-(d-b)\mathbf{N}. \end{aligned}$$

From (4), we have

5 $$\begin{aligned} \dot{\mathbf{N}}(t)+(d-b)\mathbf{N}\leq a. \end{aligned}$$

On solving (5), we have

6 $$\begin{aligned} \mathbf{N}(t)\leq \frac{a}{d-b}+C e^{-(d-b)t}, \end{aligned}$$

where *C* is a constant of integration, we see that as $t\rightarrow \infty $ in (6), then one has the required result:

$$ \mathbf{N}(t)\leq \frac{a}{d-b},\quad d>b. $$

 □

Before analyzing any biological model, it is natural to ask whether such a dynamical problem really exists or not. This question is guaranteed by fixed point theory. Here, we will try to use the same theory for the proposed problem (1) being part of this research. Regarding the aforesaid need, we express the right-hand sides of model (1) as follows:

7 $$\begin{aligned}& \mathrm{f}_{1}(t,P, I, R)= a -\sigma I(t)P(t) \bigl(1+\gamma I(t) \bigr) -dP(t) + \alpha R(t), \\& \mathrm{f}_{2}(t,P, I, R)=\sigma I(t)P(t) \bigl(1+\gamma I(t)\bigr)-( \mu +d+ \delta -b)I(t), \\& \mathrm{f}_{3}(t,P, I, R)=\mu I(t) -(\alpha +d)R(t). \end{aligned}$$

With the help of (7), the developed system can be written in the following form:

8 $$\begin{aligned}& {}^{\mathcal{ABC}}\mathrm{D}_{+0}^{\kappa }\mathcal{V}(t)=\Phi \bigl(t, \mathcal{V}(t)\bigr), \quad t\in \mathcal{J}, 0< \kappa \leq 1, \\& \mathcal{V}(0)=\mathcal{V}_{0}. \end{aligned}$$

In view of Lemma 2.3, (8) yields

9 $$ \mathcal{V}(t)=\mathcal{V}_{0}+\Phi \bigl(t, \mathcal{V}(t)\bigr) \frac{(1-\kappa )}{\mathcal{ABC}(\kappa )}+ \frac{\kappa }{\mathcal{ABC}(\kappa )\Gamma (\kappa )} \int _{0}^{t} (t- \theta )^{\kappa -1} \Phi \bigl( \theta , \mathcal{V}(\theta )\bigr)\,d\theta , $$

where

10 $$ \mathcal{V}(t)= \textstyle\begin{cases} P(t) \\ I(t) \\ R(t), \end{cases}\displaystyle \qquad \mathcal{V}_{0}= \textstyle\begin{cases} P_{0} \\ I_{0} \\ R_{0}, \end{cases}\displaystyle \qquad \Phi \bigl(t, \mathcal{V}(t)\bigr)= \textstyle\begin{cases} \mathrm{f}_{1}(t,P, I, R) \\ \mathrm{f}_{2}(t,P, I, R) \\ \mathrm{f}_{3}(t,P, I, R). \end{cases} $$

Due to (9) and (10), we define the two operators **F**, **G** from (9):

11 $$\begin{aligned}& \mathbf{F}(\mathcal{V})= \mathcal{V}_{0}+\Phi \bigl(t, \mathcal{V}(t) \bigr) \frac{(1-\kappa )}{\mathcal{ABC}(\kappa )}, \\& \mathbf{G}(\mathcal{V})= \frac{\kappa }{\mathcal{ABC}(\kappa )\Gamma (\kappa )} \int _{0}^{t} (t- \theta )^{\kappa -1}\Phi \bigl( \theta , \mathcal{V}(\theta )\bigr)\,d\theta . \end{aligned}$$

To go ahead, the given hypotheses may hold:

(H1): Let there be some constants $C_{\Phi }$, $D_{\Phi }$ with
  $$ \bigl\vert \Phi \bigl(t, \mathcal{V}(t)\bigr) \bigr\vert \leq C_{\Phi } \vert \mathcal{V} \vert +D_{\Phi }. $$
(H2): There exists constant $L_{\Phi }>0$ such that, for each $\mathcal{V}, \overline{\mathcal{V}}\in \mathbf{Z}$ such that
  $$ \bigl\vert \Phi (t, \mathcal{V})-\Phi (t, \overline{\mathcal{V}}) \bigr\vert \leq L_{\Phi }\bigl[ \vert \mathcal{V} \vert - \vert \overline{ \mathcal{V}} \vert \bigr]. $$

### Theorem 3.2

*Under hypotheses* $(H1)$, $(H2)$, *problem* (9) *has at least one solution if* $\frac{L_{\Phi }}{\mathcal{ABC}(\kappa )}<1$. *Therefore the considered model* (1) *has at least one solution*.

### Proof

We prove the theorem in two steps as follows.

*Step I*: Let $\overline{\mathcal{V}}\in \mathbf{B}$, where $\mathbf{B}=\{\mathcal{V}\in \mathbf{Z}: \Vert \mathcal{V} \Vert \leq \rho , \rho >0\}$ is a closed convex set. Then, using the definition of **F** in (11), one gets

12 $$\begin{aligned} \bigl\Vert \mathbf{F}(\mathcal{V})-\mathbf{F}(\overline{ \mathcal{V}}) \bigr\Vert =& \frac{(1-\kappa )}{\mathcal{ABC}(\kappa )}\max_{t\in \mathcal{J}} \bigl\vert \Phi \bigl(t, \mathcal{V}(t)\bigr)-\Phi \bigl(t, \overline{ \mathcal{V}}(t)\bigr) \bigr\vert , \\ \leq & \frac{L_{\Phi }}{\mathcal{ABC}(\kappa )} \Vert \mathcal{V}- \overline{\mathcal{V}} \Vert . \end{aligned}$$

Hence **F** is a contraction.

*Step II*: For the relative compactness of **G**, we show that **G** is bounded and equicontinuous.

The continuity of **G** is obvious because Φ is continuous, and also, for any $\mathcal{V}\in \mathbf{B}$, we have

13 $$\begin{aligned} \bigl\Vert \mathbf{G}(\mathcal{V}) \bigr\Vert =&\max _{t\in \mathcal{J}} \biggl\vert \frac{\kappa }{\mathcal{ABC}(\kappa )\Gamma (\kappa )} \int _{0}^{t} (t- \theta )^{\kappa -1}\Phi \bigl( \theta , \mathcal{V}(\theta )\bigr)\,d\theta \biggr\vert , \\ \leq & \frac{\kappa }{\mathcal{ABC}(\kappa )\Gamma (\kappa )} \int _{0}^{ \tau }(\tau -\theta )^{\kappa -1} \bigl\vert \Phi \bigl(\theta , \mathcal{V}(\theta )\bigr) \bigr\vert \,d \theta , \\ \leq &\frac{\tau ^{\kappa }}{\mathcal{ABC}(\kappa )\Gamma (\kappa )}[C_{ \Phi }\rho +D_{\Phi }]. \end{aligned}$$

Hence (13) guarantees the boundedness of **G**, also let $t_{1}>t_{2} \in \mathcal{J}$, then

14 $$\begin{aligned} \bigl\vert \mathbf{G}(\mathcal{V}(t_{1})-\mathbf{G}( \mathcal{V}(t_{2}) \bigr\vert =&\frac{\kappa }{\mathcal{ABC}(\kappa )\Gamma (\kappa )} \biggl\vert \int _{0}^{t_{1}}(t_{1}-\theta )^{\kappa -1}\Phi \bigl(\theta , \mathcal{V}(\theta )\bigr)\,d\theta \\ &{}- \int _{0}^{t_{2}}(t_{2}-\theta )^{ \kappa -1}\Phi \bigl(\theta , \mathcal{V}(\theta )\bigr)\,d\theta \biggr\vert , \\ \leq &\frac{[C_{\Phi }\rho +D_{\Phi }]}{\mathcal{ABC}(\kappa )\Gamma (\kappa )}\bigl[t_{1}^{\kappa }-t_{2}^{\kappa }+2(t_{2}-t_{1})^{\kappa } \bigr]. \end{aligned}$$

The right-hand side in (14) becomes zero at $t_{1}\rightarrow t_{2}$. Since **G** is continuous,

$$ \bigl\vert \mathbf{G}(\mathcal{V}(t_{1})-\mathbf{G}( \mathcal{V}(t_{1}) \bigr\vert \rightarrow 0, \quad \text{as } t_{1}\rightarrow t_{2}. $$

Since **G** is a bounded operator and continuous as well, therefore **G** is uniformly continuous and bounded. All the conditions of Theorem 2.4 hold, and so (9) has at least one solution, which means that the considered model has at least one solution. □

For uniqueness, we give the given result.

### Theorem 3.3

*Under hypothesis* $(H2)$, *problem* (9) *has a unique solution if* $\frac{[\Gamma (\kappa )+\tau ^{\kappa }]L_{\Phi }}{\Gamma (\kappa )\mathcal{ABC}(\kappa )}<1$, *and hence model* (1) *also obeys the same condition*.

### Proof

Let the operator $\mathbf{T}:\mathbf{Z}\rightarrow \mathbf{Z}$ be defined by

15 $$\begin{aligned} \begin{aligned}[b] \mathbf{T}\mathcal{V}(t)={}&\mathcal{V}_{0}+ \Phi \bigl(t, \mathcal{V}(t)\bigr) \frac{(1-\kappa )}{\mathcal{ABC}(\kappa )} \\ &\quad {} + \frac{\kappa }{\mathcal{ABC}(\kappa )\Gamma (\kappa )} \int _{0}^{t} (t- \theta )^{\kappa -1} \Phi \bigl( \theta , \mathcal{V}(\theta )\bigr)\,d\theta , \quad t\in \mathcal{J}. \end{aligned} \end{aligned}$$

Let $\mathcal{V}, \overline{\mathcal{V}} \in \mathbf{Z}$, then one can take

16 $$\begin{aligned} \Vert \mathbf{T}\mathcal{V}-\mathbf{T}\overline{\mathcal{V}} \Vert \leq &\frac{(1-\kappa )}{\mathcal{ABC}(\kappa )} \max_{t\in \mathcal{J}} \bigl\vert \Phi \bigl(t, \mathcal{V}(t)\bigr)-\Phi \bigl(t, \overline{\mathcal{V}}(t)\bigr) \bigr\vert , \\ &{}+\frac{\kappa }{\mathcal{ABC}(\kappa )\Gamma (\kappa )}\max_{t\in \mathcal{J}} \biggl\vert \int _{0}^{t} (t-\theta )^{\kappa -1} \Phi \bigl( \theta , \mathcal{V}(\theta )\bigr)\,d\theta \\ &{} - \int _{0}^{t} (t-\theta )^{\kappa -1} \Phi \bigl( \theta , \overline{\mathcal{V}}(\theta )\bigr)\,d\theta \biggr\vert , \\ \leq &\Xi \Vert \mathcal{V}-\overline{\mathcal{V}} \Vert , \end{aligned}$$

where

17 $$\begin{aligned} \Xi = \frac{[\Gamma (\kappa )+\tau ^{\kappa }]L_{\Phi }}{\Gamma (\kappa )\mathcal{ABC}(\kappa )}. \end{aligned}$$

Hence, **T** is a contraction from (16). Thus, the integral equation (9) has a unique solution and so does system (1). □

Next, to develop and present some results on stability of the problem, we will consider a small perturbation $\phi \in C(\mathcal{J})$ which depends only on the solution and $\phi (0)=0$. Further

- $\vert \phi (t) \vert \leq \varepsilon $ for $\varepsilon >0$;
- ${}^{\mathcal{ABC}}\mathrm{D}_{+0}^{\kappa }\mathcal{V}(t)=\Phi (t, \mathcal{V}(t))+\phi (t)$.

### Lemma 3.4

*The solution of the perturbed problem*

18 $$\begin{aligned}& {}^{\mathcal{ABC}}\mathrm{D}_{+0}^{\kappa }\mathcal{V}(t)=\Phi \bigl(t, \mathcal{V}(t)\bigr)+\phi (t), \\& \mathcal{V}(0)=\mathcal{V}_{0} \end{aligned}$$

*satisfies the following relation*:

19 $$\begin{aligned}& \biggl\vert \mathcal{V}(t)- \biggl(\mathcal{V}_{0}+\Phi \bigl(t, \mathcal{V}(t)\bigr) \frac{(1-\kappa )}{\mathcal{ABC}(\kappa )}+ \frac{\kappa }{\mathcal{ABC}(\kappa )\Gamma (\kappa )} \int _{0}^{t} (t- \theta )^{\kappa -1} \Phi \bigl( \theta , \mathcal{V}(\theta )\bigr)\,d\theta \biggr) \biggr\vert , \\& \quad \leq \frac{\Gamma (\kappa )+ \tau ^{\kappa }}{\mathcal{ABC}(\kappa )\Gamma (\kappa )} \varepsilon =\Omega _{\tau , \kappa }\varepsilon . \end{aligned}$$

### Proof

The proof is straightforward so we omit it. □

### Theorem 3.5

*Under assumption* (H2) *together with result* (19) *in Lemma *3.4, *the solution of integral equation* (9) *is Ulam–Hyers stable*. *Consequently*, *the analytical results of the considered system are Ulam–Hyers stable if* $\Xi <1$, *where* Ξ *is given in* (17).

### Proof

Let $\mathcal{V}\in \mathbf{Z}$ be any solution and $\overline{\mathcal{V}}\in \mathbf{Z}$ be at most one solution of (9), then

$$\begin{aligned}& \bigl\vert \mathcal{V}(t)-\overline{\mathcal{V}}(t) \bigr\vert \\& \quad = \biggl\vert \mathcal{V}(t)- \biggl(\mathcal{V}_{0}+\Phi \bigl(t, \overline{\mathcal{V}}(t)\bigr) \frac{(1-\kappa )}{\mathcal{ABC}(\kappa )}+ \frac{\kappa }{\mathcal{ABC}(\kappa )\Gamma (\kappa )} \int _{0}^{t} (t- \theta )^{\kappa -1} \Phi \bigl( \theta , \overline{\mathcal{V}}(\theta )\bigr)\,d \theta \biggr) \biggr\vert \\& \quad \leq \biggl\vert \mathcal{V}(t)- \biggl(\mathcal{V}_{0}+\Phi \bigl(t, \mathcal{W}(t)\bigr)\frac{(1-\kappa )}{\mathcal{ABC}(\kappa )}+ \frac{\kappa }{\mathcal{ABC}(\kappa )\Gamma (\kappa )} \int _{0}^{t} (t- \theta )^{\kappa -1} \Phi \bigl( \theta , \mathcal{W}(\theta )\bigr)\,d\theta \biggr) \biggr\vert \\& \quad\quad {}+ \biggl\vert \biggl(\mathcal{V}_{0}+\Phi \bigl(t, \mathcal{W}(t)\bigr) \frac{(1-\kappa )}{\mathcal{ABC}(\kappa )}+ \frac{\kappa }{\mathcal{ABC}(\kappa )\Gamma (\kappa )} \int _{0}^{t} (t- \theta )^{\kappa -1} \Phi \bigl( \theta , \mathcal{W}(\theta )\bigr)\,d\theta \biggr) \\& \quad\quad {}- \biggl(\mathcal{V}_{0}+ \Phi \bigl(t, \overline{\mathcal{V}}(t) \bigr) \frac{(1-\kappa )}{\mathcal{ABC}(\kappa )}+ \frac{\kappa }{\mathcal{ABC}(\kappa )\Gamma (\kappa )} \int _{0}^{t} (t- \theta )^{\kappa -1} \Phi \bigl( \theta , \overline{\mathcal{V}}(\theta )\bigr)\,d \theta \biggr) \biggr\vert \\& \quad \leq \Omega _{\tau , \kappa }\varepsilon + \frac{L_{\Phi }}{\mathcal{ABC}(\kappa )} \Vert \mathcal{V}- \overline{\mathcal{V}} \Vert + \frac{\tau ^{\kappa }L_{\Phi }}{\mathcal{ABC}(\kappa )\Gamma (\kappa )} \Vert \mathcal{V}-\overline{\mathcal{V}} \Vert , \end{aligned}$$

from which we have

20 $$\begin{aligned} \Vert \mathcal{V}-\overline{\mathcal{V}} \Vert \leq \Omega _{\tau , \kappa } \varepsilon +\Xi \Vert \mathcal{V}- \overline{\mathcal{V}} \Vert . \end{aligned}$$

From (20), we can write

21 $$ \Vert \mathcal{V}-\overline{\mathcal{V}} \Vert \leq \frac{\Omega _{\tau , \kappa }}{1-\Xi }\varepsilon . $$

Hence result (21) concludes that the solution of (9) is Ulam–Hyers stable and, consequently, the solution of the considered system is Ulam–Hyers stable. □

## Construction of numerical algorithm for the constructed model (1)

This section is devoted to numerical results. Here we use a coupled numerical method due to the combination of fundamental theorem of fractional calculus and the two-step Lagrange polynomial as used in [65]. From the first equation of the model under our consideration, in view of (7), we let

22 $$\begin{aligned}& {}^{\mathcal{ABC}}\mathrm{D}_{+0}^{\kappa }{P}(t)= \mathrm{f}_{1}\bigl(t, P(t), I(t), R(t)\bigr), \\& P(0)=P_{0}. \end{aligned}$$

In view of Lemma 2.3, (22) implies that

23 $$ \begin{aligned}[b] P(t)={}&P_{0}\frac{1-\kappa }{\mathcal{ABC}(\kappa )}\mathrm{f}_{1} \bigl(t, P(t), I(t), R(t)\bigr) \\ &{} +\frac{\kappa }{\mathcal{ABC}(\kappa )} \frac{1}{\Gamma (\kappa )} \int _{0}^{t}(t-\theta )^{\kappa -1} \mathrm{f}_{1}\bigl(\theta , P(\theta ), I(\theta ), R(\theta )\bigr)\,d \theta .\end{aligned} $$

Now, in terms of Lagrange interpolation polynomials, we may write over $[t_{k}, t_{k+1}]$ the function $\mathrm{f}_{1}(\theta , P(\theta ), I(\theta ), R(\theta ))$ with $\mathrm{h}=t_{k}-t_{k-1}$:

24 $$\begin{aligned} \begin{aligned}[b] \mathbf{P}_{k}\approx {}&\frac{1}{\mathrm{h}} \bigl[(\theta -t_{k-1}) \mathrm{f}_{1}\bigl(t_{k}, P(t_{k}), I(t_{k}), R(t_{k})\bigr) \\ &{}-(\theta -t_{k}) \mathrm{f}_{1}\bigl(t_{k-1}, P(t_{k-1}), I(t_{k-1}), R(t_{k-1})\bigr) \bigr]. \end{aligned} \end{aligned}$$

Plugging (24) in (23), we may write (23) as follows:

25 $$\begin{aligned} P(t_{n+1}) =&P_{0}+ \frac{(1-\kappa ) }{\mathcal{ABC}(\kappa )} \mathrm{f}_{1}\bigl(t_{k}, P(t_{k}), I(t_{k}), R(t_{k})\bigr) \\ & {} +\frac{\kappa }{\mathcal{ABC}(\kappa )\Gamma (\kappa )}\sum_{j=0}^{n} \biggl( \frac{\mathrm{f}_{1}(t_{j}, P(t_{j}), I(t_{j}), R(t_{j}))}{\mathrm{h} }\int _{t_{j}}^{t_{j+1}}(\theta -t_{j-1}) (t_{n+1}-\theta )^{\kappa -1}\,d \theta \\ & {} - \frac{\mathrm{f}_{1}(t_{j-1}, P(t_{j-1}), I(t_{j-1}), R(t_{j-1}))}{\mathrm{h} }\int _{t_{j}}^{t_{j+1}}(\theta -t_{j}) (t_{n+1}-\theta )^{\kappa -1}\,d \theta \biggr) \\ =&P_{0}+\frac{(1-\kappa ) }{\mathcal{ABC}(\kappa )}\mathrm{f}_{1} \bigl(t_{n},P(t_{n}), I(t_{n}), R(t_{n})\bigr) \\ & {} +\frac{\kappa }{\mathcal{ABC}(\kappa )\Gamma (r)}\sum_{j=0}^{n} \biggl( \frac{\mathrm{f}_{1}(t_{j}, P(t_{j}), I(t_{j}), R(t_{j}))}{\mathrm{h} } \Omega _{j-1,\kappa } \\ & {}- \frac{\mathrm{f}_{1}(t_{j-1}, P(t_{j-1}), I(t_{j-1}), Q(t_{j-1}))}{\mathrm{h} } \Lambda _{j,\kappa } \biggr), \end{aligned}$$

where the notions $\Omega _{j-1,\kappa }$ and $\Lambda _{j,\kappa }$ are given as follows:

26 $$\begin{aligned} \Omega _{j-1,\kappa } =& \int _{t_{j}}^{t_{j+1}} ( \theta -t_{j-1} ) ( t_{n+1}-\theta ) ^{\kappa -1}\,d\theta \\ =& -\frac{1}{\kappa } \bigl[ ( t_{j+1}-t_{j-1} ) ( t_{n+1}-t_{j+1} ) ^{\kappa }- ( t_{j}-t_{j-1} ) ( t_{n+1}-t_{j} ) ^{\kappa } \bigr] \\ & {} -\frac{1}{\kappa ( \kappa +1 ) } \bigl[ ( t_{n+1}-t_{j+1} ) ^{\kappa +1}- ( t_{n+1}-t_{j} ) ^{\kappa +1} \bigr] \end{aligned}$$

and

27 $$\begin{aligned} \Lambda _{j,r } =& \int _{t_{j}}^{t_{j+1}} (\theta -t_{j} ) ( t_{n+1}-\theta ) ^{\kappa -1}\,d\theta \\ =&-\frac{1}{\kappa } \bigl[ ( t_{j+1}-t_{j} ) ( t_{n+1}-t_{j+1} ) ^{\kappa } \bigr] \\ &{}- \frac{1}{\kappa ( \kappa +1 ) } \bigl[ ( t_{n+1}-t_{j+1} ) ^{\kappa +1}- ( t_{n+1}-t_{j} ) ^{\kappa +1} \bigr]. \end{aligned}$$

Put $t_{j}=j\mathrm{h} $ in (26) and (27), one has

28 $$\begin{aligned} \Omega _{j-1,\kappa } =&-\frac{\mathrm{h} ^{\kappa +1}}{\kappa } \bigl[ \bigl( j+1- ( j-1 ) \bigr) \bigl( n+1- ( j+1 ) \bigr) ^{\kappa }- \bigl( j- ( j-1 ) \bigr) ( n+1-j ) ^{\kappa } \bigr] \\ & {} -\frac{\mathrm{h} ^{\kappa +1}}{\kappa ( \kappa +1 ) } \bigl[ \bigl( n+1- ( j+1 ) \bigr) ^{\kappa +1}- ( n+1-j ) ^{\kappa +1} \bigr] \\ =&\frac{\mathrm{h} ^{\kappa +1}}{\kappa ( \kappa +1 ) } \bigl[ -2 ( \kappa +1 ) ( n-j ) ^{\kappa }+ (\kappa +1 ) ( n+1-j ) ^{\kappa }- ( n-j ) ^{\kappa +1}+ ( n+1-j ) ^{\kappa +1} \bigr] \\ =&\frac{\mathrm{h} ^{\kappa +1}}{\kappa ( r +1 ) } \bigl[ ( n-j ) ^{\kappa } \bigl( -2 ( \kappa +1 ) - ( n-j ) \bigr) + ( n+1-j ) ^{\kappa } ( \kappa +1+n+1-j ) \bigr] \\ =&\frac{\mathrm{h} ^{\kappa +1}}{\kappa (\kappa +1 ) } \bigl[ ( n+1-j ) ^{\kappa } ( n-j+2+\kappa ) - ( n-j ) ^{\kappa } ( n-j+2+2\kappa ) \bigr] \end{aligned}$$

and

29 $$\begin{aligned} \Lambda _{j,\kappa } =&-\frac{\mathrm{h} ^{\kappa +1}}{\kappa } \bigl[ ( j+1-j ) \bigl( n+1- ( j+1 ) \bigr) ^{ \kappa } \bigr] \\ &{}- \frac{h^{\kappa +1}}{\kappa (\kappa +1 ) } \bigl[ \bigl( n+1- ( j+1 ) \bigr) ^{\kappa +1}- ( n+1-j ) ^{ \kappa +1} \bigr] \\ =&\frac{\mathrm{h} ^{\kappa +1}}{\kappa (\kappa +1 ) } \bigl[ - ( \kappa +1 ) ( n-j ) ^{\kappa }- ( n-j ) ^{\kappa +1}+ ( n+1-j ) ^{\kappa +1} \bigr] \\ =&\frac{\mathrm{h} ^{\kappa +1}}{\kappa (\kappa +1 ) } \bigl[ ( n-j ) ^{\kappa } \bigl( - ( \kappa +1 ) - ( n-j ) \bigr) + ( n+1-j ) ^{ \kappa +1} \bigr] \\ =&\frac{\mathrm{h} ^{\kappa +1}}{\kappa ( \kappa +1 ) } \bigl[ ( n+1-j ) ^{\kappa +1}- ( n-j ) ^{ \kappa } ( n-j+1+\kappa ) \bigr] . \end{aligned}$$

Substituting (28) and (29) into (25), we get

30 $$\begin{aligned} P(t_{n+1}) =&P(t_{0})+ \frac{1-\kappa }{\mathcal{ABC}(\kappa )} \mathrm{f}_{1}\bigl(t_{n},P(t_{n}), I(t_{n}), R(t_{n})\bigr)+ \frac{\kappa }{\mathcal{ABC}(\kappa )} \\ & {}\times\sum _{j=0}^{n}\biggl( \frac{\mathrm{f}_{1}(t_{j}, P(t_{j}), I(t_{n}), R(t_{n}) )}{\Gamma (r +2)} \\ & {}\times\mathrm{h}^{\kappa } \bigl[ (n+1-j)^{\kappa }(n-j+2+\kappa )-(n-j)^{\kappa }(n-j+2+2 \kappa ) \bigr] \\ & {} - \frac{\mathrm{f}_{1}(t_{j-1}, P(t_{j-1}), I(t_{j-1}), Q(t_{j-1}))}{\Gamma (\kappa +2)} \mathrm{h} ^{\kappa } \bigl[ (n+1-j)^{\kappa +1}-(n-j)^{\kappa }(n-j+1+ \kappa ) \bigr] \biggr). \end{aligned}$$

Similarly,

31 $$\begin{aligned} I(t_{n+1}) =&I(t_{0})+ \frac{1-\kappa }{\mathcal{ABC}(\kappa )} \mathrm{f}_{2}\bigl(t_{n},P(t_{n}), I(t_{n}), R(t_{n})\bigr)+\frac{\kappa }{\mathcal{ABC}(\kappa )} \\ &{}\times \sum _{j=0}^{n} \biggl( \frac{\mathrm{f}_{1}(t_{j-1}, P(t_{j-1}), I(t_{j-1}), R(t_{j-1}))}{\Gamma (\kappa +2)} \\ & {}\times\mathrm{h} ^{\kappa } \bigl[ (n+1-j)^{\kappa }(n-j+2+\kappa )-(n-j)^{\kappa }(n-j+2+2 \kappa ) \bigr] \\ & {} - \frac{\mathrm{f}_{2}(t_{j-1}, P(t_{j-1}), I(t_{j-1}), R(t_{j-1}))}{\Gamma (\kappa +2)} \mathrm{h} ^{\kappa } \bigl[ (n+1-j)^{\kappa +1}-(n-j)^{\kappa }(n-j+1+ \kappa ) \bigr] \biggr) \end{aligned}$$

and

32 $$\begin{aligned} R(t_{n+1}) =&R(t_{0})+ \frac{1-\kappa }{\mathcal{ABC}(\kappa )} \mathrm{f}_{3}\bigl(t_{n}, P(t_{n}), I(t_{n}), R(t_{n})\bigr)+\frac{r }{\mathcal{ABC}(\kappa )} \\ &{}\times \sum_{j=0}^{n} \biggl( \frac{\mathrm{f}_{3}(t_{j-1}, P(t_{j-1}), I(t_{j-1}), R(t_{j-1}))}{\Gamma (\kappa +2)} \\ & {}\times\mathrm{h} ^{\kappa } \bigl[ (n+1-j)^{\kappa }(n-j+2+\kappa )-(n-j)^{\kappa }(n-j+2+2 \kappa ) \bigr] \\ & {} - \frac{\mathrm{f}_{3}(t_{j-1}, P(t_{j-1}), I(t_{j-1}), R(t_{j-1}))}{\Gamma (\kappa +2)} \mathrm{h} ^{\kappa } \bigl[ (n+1-j)^{\kappa +1}-(n-j)^{\kappa }(n-j+1+ \kappa ) \bigr] \biggr). \end{aligned}$$

## Numerical interpretation and discussion

In this section, we compute approximate solutions by using some real values of the parameters for the considered model as given in Table 2. We took some initial population of susceptible, infected, and recovered class as $11, 0.084, 0$ in millions, respectively. Death from infection or natural is taken as 0.02 during first 60 days.

**Table 2:** Description of the parameters used in model (1) and their numerical values

Parameters	The physical interpretation	Numerical value
*P*(*t*)	Susceptible compartment [64]	11 in millions
*I*(*t*)	Infected compartment [64]	0.084 in millions
*R*(*t*)	Recovered compartment due to death	0 millions
*a*	The recruitment rate (assumed)	0.00073
*d*	Natural death [6, 64]	0.02
*δ*	Death due to corona [64]	0.0000357
*b*	The immigration rate of infected individuals	0.000001, 0.00791
*μ*	Infected population goes to removed [64]	0.00047876
*σ*	The infection rate [6]	0.580
*γ*	The rate at which recovered individuals lose immunity	0.00197
*α*	The recovery rate [64]	0.09871

**Figure 1:**
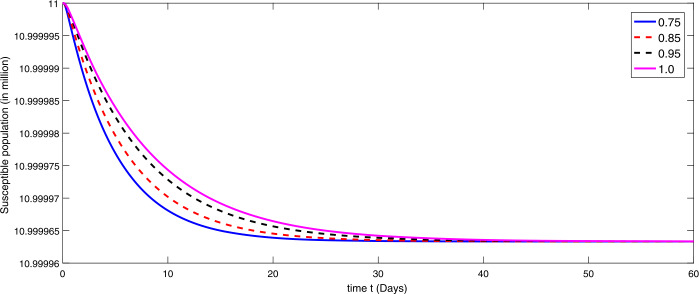
The plot shows the dynamics of the susceptible class in model (1) at various values of fractional order *κ* using $\mathcal{ABC}$ derivative at the minimum value of immigration rate

**Figure 2:**
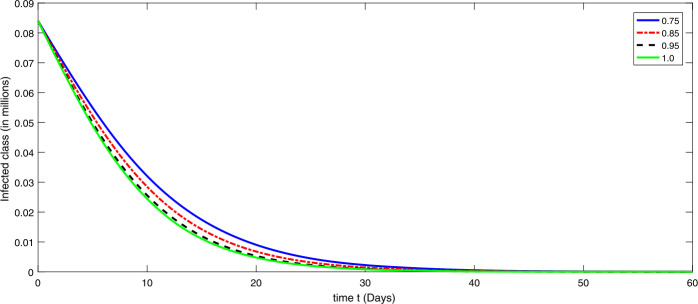
The plot shows the dynamics of the infected class at various values of fractional order *κ* using $\mathcal{ABC}$ derivative at the minimum value of immigration rate

**Figure 3:**
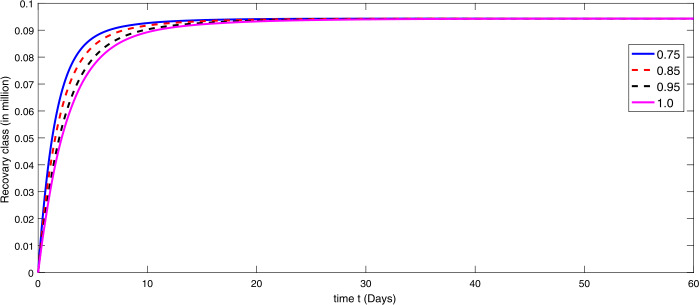
The dynamics of the recovered class at various values of fractional order *κ* using $\mathcal{ABC}$ derivative at the minimum value of immigration rate

**Figure 4:**
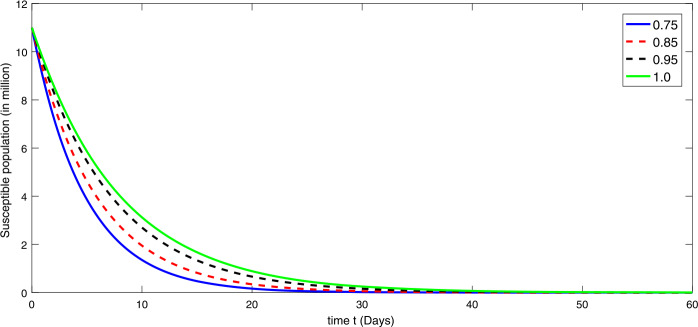
The plot shows the dynamics of the susceptible class in model (1) at various values of fractional order *κ* using $\mathcal{ABC}$ derivative in the presence of the maximum value of immigration rate

**Figure 5:**
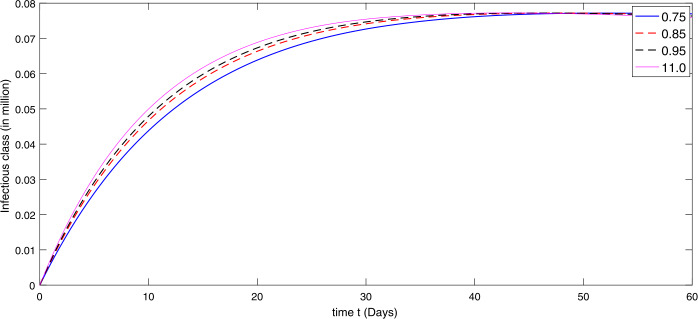
The plot shows the dynamics of the infected class in model (1) at various values of fractional order *κ* using $\mathcal{ABC}$ derivative in the presence of the maximum value of immigration rate

**Figure 6:**
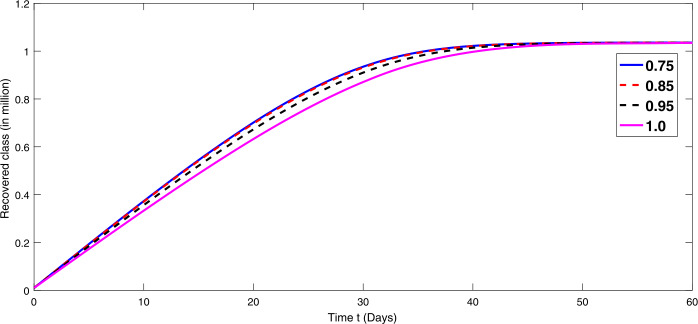
The plot shows the dynamics of the recovered class in model (1) at various values of fractional order *κ* using $\mathcal{ABC}$ derivative in the presence of maximum value of immigration rate

By using the parameter values in Table 2, we simulate the results by using Matlab subject to the above algorithms for various compartments in (30), (31), and (32) as in Figures 1–3. First we consider that there is minimum immigration that is $b=0.000001$. Then, from Figures 1–3, we see that the infection as well as the removed due to death of coronavirus are decreasing at different rate due to fractional order derivatives. The lower the rate the faster the decay rate and vice versa. From Figures 1–3, on minimizing the immigration of people in Wuhan city, the infection has been controlled in nearly sixty days. The population of susceptible people will go on increasing, and consequently the other compartments which are infection and death will go on decreasing at different rate. Since fractional order derivative provides greater degree of freedom, the dynamics of grow and decay is different. It is slightly faster at smaller order to approach a stable position as compared to larger order. Next, in Figures 4–6, we checked the effect on assumed maximum immigration rate $b=0.000791$ for the given population and simulated the results. We see from Figures 4–6 that when immigration is increasing the susceptible population goes on on deceasing with different order due to fractional derivative. It is faster on smaller order and vice versa. Also the infected and recovered population are increasing as more people will catch infection and hence more deaths will occur. The increase in the population of recovered class is shown in Figures 4–6.

## Conclusion

This manuscript has studied a new type model for COVID-19 under nonsingular kernel-type derivative. First of all we have proved the feasible region and boundedness of the model. Then we have established the results for the existence of such a model in the real world by using the fixed point theory of Banach and Krasnoselskii. Also we have established necessary conditions for Ulam–Hyers stability via nonlinear functional analysis. By applying the fractional-type AB method, we have simulated the results and shown that immigration has great impact on transmission dynamics of the current outbreak. Adopting precautionary measures including minimum immigration will reduce the transmission of the disease in a society. Also, for such type of a dynamical study, fractional calculus may be used as a powerful tool to understand the global dynamics of the mentioned disease. As compared to the local fractional derivative, the ABC derivative of arbitrary order is nonlocal and nonsingular, which may produce more significant results in many situations.
